# Protective Effects of Oxyresveratrol in Isoproterenol-Induced Myocardial Infarction in Rats: A Stereological Study*

**DOI:** 10.5152/eurasianjmed.2024.23214

**Published:** 2024-09-13

**Authors:** Huseyn Aliyev, Sibel Bilgili, Erdem Toktay, Nubar Nuriyeva, Yasin Bayir

**Affiliations:** 1 Department of Pharmacuitical Chemistry, Azerbaijan Medical University Faculty of Pharmacy, Baku, Azerbaijan; 2 Department of Mathematics Education, Atatürk University Faculty of Education, Erzurum, Türkiye; 3 Department of Histology and Embryology, Kafkas University Faculty of Medicine, Kars, Türkiye; 4 Department of Biochemistry, Atatürk University Faculty of Pharmacy, Erzurum, Türkiye

**Keywords:** Antioxidants, cardioprotective, myocardial infarction, oxyresveratrol, rat

## Abstract

**Background:**

The aim of this study is to examine the protective effect of oxyresveratrol (OXY) against isoproterenol-induced myocardial infarction in rats, through routine biochemical parameters and oxidative stress parameters that show heart damage.

**Methods:**

Oxyresveratrol was administered by oral gavage at doses of 10 and 20 mg/kg, respectively, once a day for 5 days. On the fourth and fifth days, 180 mg/kg isoproterenol was administered intraperitoneally to the OXY treatment group and control groups. Twenty-four hours after the last isoproterenol application, blood and heart tissue were taken under anesthesia and transferred to −80 degrees and formalin for biochemical and histopathological studies. CK-MB and TnI levels were measured in serum obtained from blood. In the heart tissue, antioxidant parameters, superoxide dismutase (SOD), glutathione (GSH) and malondialdehyde (MDA) levels, and histopathological and stereological evaluations were performed.

**Results:**

Oxyresveratrol has antioxidant and anti-inflammatory activity depending on the dose. Oxyresveratrol showed potent protective effect against isoproterenol-induced myocardial infarction. It has been proven that at all doses of oxyresveratrol, statistically, isoproterenol decreased the MDA level, which was one of the oxidative stress markers, compared to the control group, and increased SOD activity and GSH levels. Similar to the biochemically determined parameters, oxyresveratrol treatment was also found to have a protective effect at the cell level, histopathologically and stereologically.

**Conclusion:**

All results show that oxyresveratrol has strong antioxidant and anti-inflammatory activity, especially at a dose of 20 mg/kg, it significantly reduces myocardial damage and this agent has a cardioprotective effect.

## Introduction

The cardiovascular system has a very important role in living organisms and circulates vital oxygen and nutrients between organs and blood. The number of deaths due to cardiovascular diseases is increasing every year, and myocardial infarction (MI) has an important place among these diseases. It is estimated that cardiovascular disease-related deaths will reach 23 million in 2030.^1^ Myocardial infarction, heart attack in other words, is a serious condition that causes tissue damage when blood flow is suddenly cut off as a result of a blockage in the coronary arteries that supply the heart muscle. It has been demonstrated by many studies that oxidative stress caused by reactive oxygen species (ROS) formed during ischemic damage contributes to the pathogenesis of MI. As far as the obstruction in the coronary artery is not dissolved, ischemia increases step by step and results in cellular necrosis or death.^2^ Myocardial infarction is one of the most frequent ischemic heart disorders, and it can cause mortality and disability globally.^3^ It is characterized by biochemical, hemodynamic, and histological abnormalities, such as increased heart rate, vascular pressure, and ventricular dysfunction, as well as depletion of endogenous antioxidants, expulsion of cardiac marker enzymes, and lipid peroxidation.^4^ These changes are the result of excess ROS such as hydroxyl radicals, superoxide anion, MDA, etc., in ischemic tissues and cause oxidative damage to membrane components such as lipids, proteins, carbohydrates, and genetic material.^5^ For these reasons, targeting oxidative damage during ischemic conditions by antioxidants is considered to be a good option in preventing the occurrence and complications of ischemic heart disease.^6^ Synthetic antioxidants can demonstrate toxic, mutagenic, and/or pro-oxidant activities that limit their usage in heart diseases; that’s why researchers have shifted their attention to naturally derived antioxidants.

As a beta-adrenergic receptor agonist, isoproterenol (ISO), which has a catecholamine structure, induces severe stress in the myocardium by stimulating the myocardium strongly, resulting in MI-like cell death and obstruction in coronary arteries at high doses.^7^ Various mechanisms put forward to explain the damage caused by ISO include tachycardia, hypoxia due to coronary hypotension,^8^ calcium overload,^9^ depletion of energy reserves,^10^ and cytotoxic ROS accumulation via auto-oxidation of catecholamine structured amines.^7^ Reactive oxygen generated as a result of increased oxygen demand causes the lipid membrane to deteriorate and the MI model to form.^11^

Since ancient times, spices and herbs have been employed for their antioxidant activity and have been found to play a key role in the treatment of many different ailments, including cardiovascular disease.^2^ During the last decades, studies focused on the importance of natural antioxidants and their sources as potential therapeutic agents. Oxyresveratrol (OXY) is the aglycone of Mulberosside A, a stilbenoid compound found in Artocarpus Lakoocha and Morus alba. Its chemical structure consists of a dihydroxylated trans-1,2-diphenylethylene structure (IUPAC name: 4-[(E)-2-(3,5- dihydroxy phenyl)-ethenyl] benzene-1,3-diol; C_14_H_12_O_4_). The antioxidant and anti-inflammatory properties of OXY, a natural stilbenoid that has attracted much attention recently with its simple chemical structure and wide pharmacological effect, have been extensively studied. Oxyresveratrol has pleiotropic effects such as inhibition of tyrosinase enzymes, decrease of melanogenesis, increase of antioxidant status, anti-inflammatory activities, and preventive roles against digestive diseases and neurological disorders.^12^ As a resveratrol derivative,^13^ OXY is obtained from the plant Artocarpus lakoocha Roxb as a natural polyphenol with good antioxidant activity.^14^ In particular, OXY protects neuron cells,^15^ human lens epithelial cells,^16^ and liver cells such as hepatocytes,^17^ and limits DNA damage by scavenging ROS products.^18^ In addition, Lee et al suggested that OXY reduces nitric oxide formation in murine macrophages, which is also an oxidant marker that can cause cell damage in many inflammatory conditions.^19^

Based on the existing literature, there appears to be no study detailing the effects of OXY on ISO-induced cardiac injury in rats. This study aimed to examine the therapeutic potential of OXY as a cardioprotective drug in an animal model of ISO-induced MI. For this purpose, biochemical analyses were performed on cardiac enzymes and oxidative markers. Also, the heart tissues of rats were evaluated by histopathological and stereological studies.

## Material and Methods

### Chemicals

All chemicals were acquired from a licensed source. Chemicals like OXY, ISO, and other substances were of analytical grade.

### Experimental Animals

In this experimental study, a cohort of thirty male Albino Wistar rats, weighing between 200 and 220 grams, was utilized. Ethical clearance for the research was obtained from the Ethical Committee of the Experimental Animal Teaching and Research Center at Atatürk University under Protocol (E-93722986-000-2100359379). The rats were procured from the Medical and Experimental Application and Research Center, located in Erzurum, Türkiye (ATADEM). They were maintained in a controlled laboratory environment, following a natural light-dark cycle, and had unrestricted access to both water and a standard diet.

### In vivo Experimental Protocol

In this research investigation, a cohort of 30 rats was divided into 5 separate groups, with each group comprising 6 rats. The specific characteristics of these experimental groups are described below:

Group 1, healthy group that received isotonic NaCl as a vehicle; Group 2, ISO control that received a 180 mg/kg dose of ISO; Group 3, healthy rats that received OXY 20 mg/kg; Group 4, low dose treated rats that received ISO (180 mg/kg) + OXY 10 mg/kg; Group 5, high dose treated rats that received ISO (180 mg/kg) + OXY 20 mg/kg.

During the first 5 days of the experiment, for low- and high-dose treatment groups, OXY 10 and 20 mg/kg were administered by oral rat gavage once a day. A 180 mg/kg dose of ISO dissolved in isotonic NaCl was administered to rats subcutaneously for the induction of myocardial injury.^7^ The healthy group (Group 1) was administered isotonic solution subcutaneously. To induce myocardial injury in the rat model, the ISO control group (Group 2) and OXY treatment groups (Groups 4 and 5) received subcutaneous injections of ISO (180 mg/kg) on days 4 and 5 of the drug treatment, with a 24-h interval between each injection.

Healthier rats in the OXY control group (Group 3) were orally administered OXY at a dose of 20 mg/kg, with a 24-h interval between each dose over a period of 5 consecutive days.

Following the experimental period, the animals were humanely euthanized by administering sodium thiopental at a dosage of 50 mg/kg. To get serum, blood samples were obtained from the animals without adding anticoagulants. The collected blood was then processed through centrifugation to separate the serum from other blood components. Furthermore, the hearts from all the rat groups were carefully excised and placed on cold-ice bars to maintain their temperature and quality. Subsequently, specific sections of the rat hearts were isolated for the purpose of conducting biochemical analyses. These portions were then transported and frozen at −80°C to maintain their biological characteristics. Additionally, the other parts of the heart tissues were placed in a solution containing 10% formaldehyde. This step was taken to prepare the heart tissues for histopathological examination, ensuring their preservation and suitability for a detailed examination of tissue structure and pathology.

### Biochemical Studies

#### Cardiac Enzyme Measurements

The serum samples, obtained following the centrifugation process, were employed to assess CK-MB activities and the concentrations of TnI using an automated analyzer (Roche Cobas e-801).^20^ The measurements were expressed as ng/mL for TnI and U/L for CK-MB.

### Assessment of Superoxide Dismutase, Glutathione , and Malondialdehyde Concentrations in Cardiac Tissue

Following the macroscopic examination, cardiac tissue homogenates were meticulously prepared to assess superoxide dismutase (SOD) enzyme activity and quantify glutathione (GSH) and lipid peroxidation concentrations using the thiobarbituric assay to measure malondialdehyde (MDA) levels in all experimental rat groups. In the biochemical section, hearts from each group of rats were subjected to cryogenic grinding with liquid nitrogen within a mortar. Then, using an Eppendorf tube and the TissueLyser II, 100 mg of finely powdered cardiac tissue was homogenized in 1 mL of phosphate-buffered saline (PBS) buffer. The homogenates were then centrifuged at 4°C in a chilled centrifuge, and the supernatants were used for the following biochemical tests. All assays were conducted at room temperature, and triplicate measurements were made for each sample. SOD enzyme activity was determined following the methodology established by Sun et al,^21^ also performed at room temperature in triplicate and was expressed as U/mg of protein. To gauge cardiac tissue lipid peroxidation, the concentration of MDA was assessed using the thiobarbituric acid reaction^22^ and the results were expressed as nmol/mg of protein. Glutathione levels within the cardiac tissue were quantified using a previously reported method^23^ and are presented as nmol/g of tissue.

### Protein Concentration Assessment

The protein concentrations in the samples were measured using the Lowry technique, utilizing commercial protein standards (Sigma Aldrich, Total protein kit-TP0300-1KT, USA).^24^ The Lowry protein assay, based on the development of a characteristic color change in the sample solution that is directly proportional to the protein concentration, is a well-known biochemical method used to measure the total protein content of a solution. This colorimetric change can be accurately measured, allowing for the quantification of total protein concentration.

### Histological Examination

All histopathological analyses were performed in the Research Laboratory of the Department of Histology and Embryology., Faculty of Medicine at Kafkas University.

At the conclusion of the experiment, tissue samples were placed in a formalin solution, and tissue follow-up was performed after 48 h of fixation. The tracking protocol is as follows: tissue samples were washed under running water for 2 h, followed by immersion in 50% (2 h), 70% alcohol (1 h), 80% alcohol (1 h), 96% alcohol (1 h), 99% alcohol (1 h). Then, 3 batches of xylene (3 × 15 min). Finally, the tissues were soaked in melted paraffin (2 × 1 h). Upon completion of the follow-up process, the tissue samples were embedded in paraffin.^25^

The blocks obtained were placed in the microtome device, and 5-µm-thick sections from each block were taken on polylysine slides. The slides were routinely stained with hematoxylin and eosin. According to this, tissue samples taken on the slide were kept in an oven at 60°C for 20 min. Then, they were passed through the xylene series 3 times for 5 min each. Afterward, the slides were kept in decreasing alcohol series (99%, 96%, 80%, 70%, 50%) for 2 min each. Nucleus staining was accomplished by immersing the samples in Harris hematoxylin dye for a duration of 3 min. The samples were stained for 2 min in the Eosin Y solution for counterstaining. Finally, the slides were passed through 96% and 99% alcohol and xylene series, and the slide surfaces were covered with a coverslip with a gluing medium.^25^

### Stereological Estimation of Vertical Heart Muscle Area Using the Stereological Nucleator Method

Estimated areas of vertical heart muscle fibers between groups were calculated using Stereo Investigator software (Microbrightfield Stereo-Investigator software 9.0; Microbrightfield). The following formula is used for calculation.^26^

Area estimate α = π*l*^2^ *I =* Length of rays

### Statistical Analysis

The statistical analysis was conducted using one-way ANOVA. As a post-hoc test, the LSD and Tukey’s multiple range tests were employed for biochemical data and the Tukey test for stereological data, both using the BMI-SPSS software package version 20.00. *P-*values <.05 were considered statistically significant. The provided results are expressed as mean values ± standard deviation (SD), using data taken from 6 rats per group.

## Results

### Biochemical Findings for Serum TnI Levels and CK-MB Activity

The impact of OXY, given at dosages of 10 mg/kg and 20 mg/kg, on cardiac indicators such as CK-MB and TnI in the serum of both normal rats and those induced with ISO is presented in Table 1. Following the induction of ISO in rats (Group 2), there was a noteworthy increase (*P* < .05) in the activity of CK-MB and levels of TnI during the acute phase when compared to the control group (Group 1). Interestingly, it was observed that in the acute treatment groups receiving OXY (ISO + OXY 10 mg/kg and 20 mg/kg), there was a significant reduction (*P* < .05) in CK-MB enzyme activity and TnI levels compared to the acute MI group (ISO Control). Among these treatment groups, the most substantial reductions in CK-MB activities and TnI levels were observed in the OXY 20 mg/kg + MI group, when compared to the OXY 10 mg/kg + MI group. Moreover, the results showed that there were no significant alterations in CK-MB enzyme activity and TnI levels in the OXY control group when compared to the intact control group. This indicates that the administration of OXY at the specified dosages in the absence of ISO-induced stress did not produce significant changes in these cardiac indicators.

### The Results Regarding Superoxide Dismutase Activities in Heart Tissue

**Figure 1.**
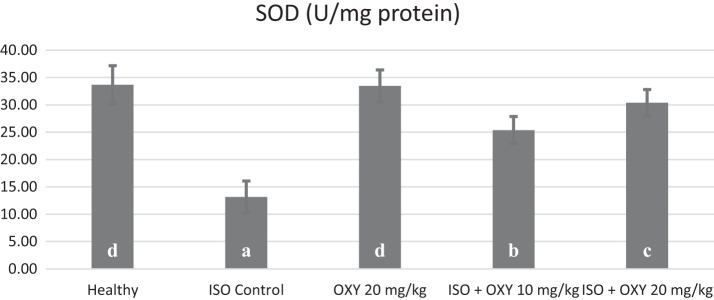
Effect of oxyresveratrol on the superoxide dismutase activities in heart tissue after isoproterenol-induced myocardial injury in rats.

Figure 1 shows the effect of OXY at dosages of 10 mg/kg and 20 mg/kg on the activity of the antioxidant enzyme SOD in the cardiac tissue of ISO-induced rats. ISO-induced rats showed significantly lower SOD activity compared to healthy controls (*P* < .05). The ISO groups pre-treated with OXY at 10 mg/kg and 20 mg/kg showed significantly higher SOD activity than the ISO control group (*P *< .05). Furthermore, the results show that SOD enzyme activity in the drug control groups was statistically unaltered when compared to the healthy control group. This implies that the substances administered in the drug control groups did not have a significant impact on SOD activity in the heart tissue, and any observed effects were likely specific to OXY treatment.

### The Results Regarding Malondialdehyde Levels in Heart Tissue

**Figure 2.**
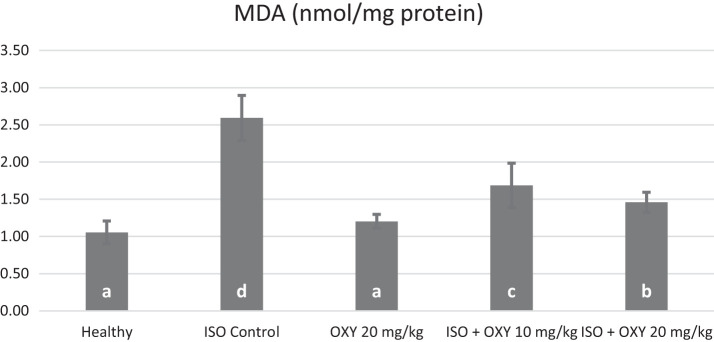
Effect of OXY on the malondialdehyde levels in heart tissue after isoproterenol-induced myocardial injury in rats.

Figure 2 shows how OXY delivered at dosages of 10 mg/kg and 20 mg/kg affected MDA levels in the cardiac tissue of rats induced by ISO. ISO-induced rats showed significantly higher MDA levels compared to the healthy control group (*P* < .05). The ISO groups that received OXY pre-treatment at 10 mg/kg and 20 mg/kg had significantly lower MDA levels than the ISO control group (*P* < .05). Furthermore, the findings show that there were no significant variations in MDA levels between the OXY and healthy groups. This implies that the administration of OXY did not lead to notable changes in MDA levels under these experimental conditions.

### The Results Regarding Glutathione Levels in Heart Tissue

**Figure 3.**
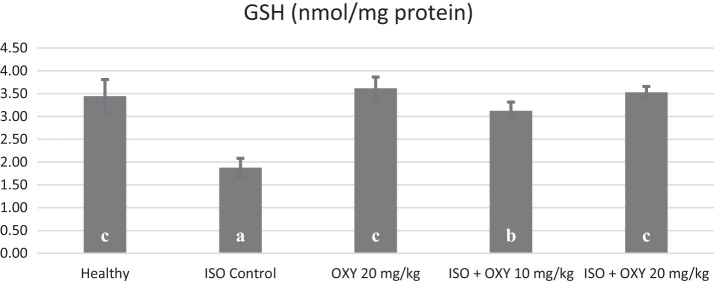
Effect of OXY on the g**lutathione** levels in heart tissue after isoproterenol-induced myocardial injury in rats.

The impact of OXY administered at doses of 10 mg/kg and 20 mg/kg on the levels of GSH in the heart tissue of rats induced with ISO is depicted in Figure 3. Remarkably, in ISO-induced rats, there was a notable decrease in GSH levels when compared to the healthy control group (*P* < .05). The ISO groups that received pre-treatment with OXY at 10 mg/kg and 20 mg/kg showed significantly higher GSH levels than the ISO control group (*P *< .05). Furthermore, the data indicate that there were no significant changes in GSH levels in the drug control group vs the healthy control group. This suggests that the drug control group did not show significant changes in GSH levels compared to the healthy control group in this investigation.

### Histological Findings

**Figure 4.**
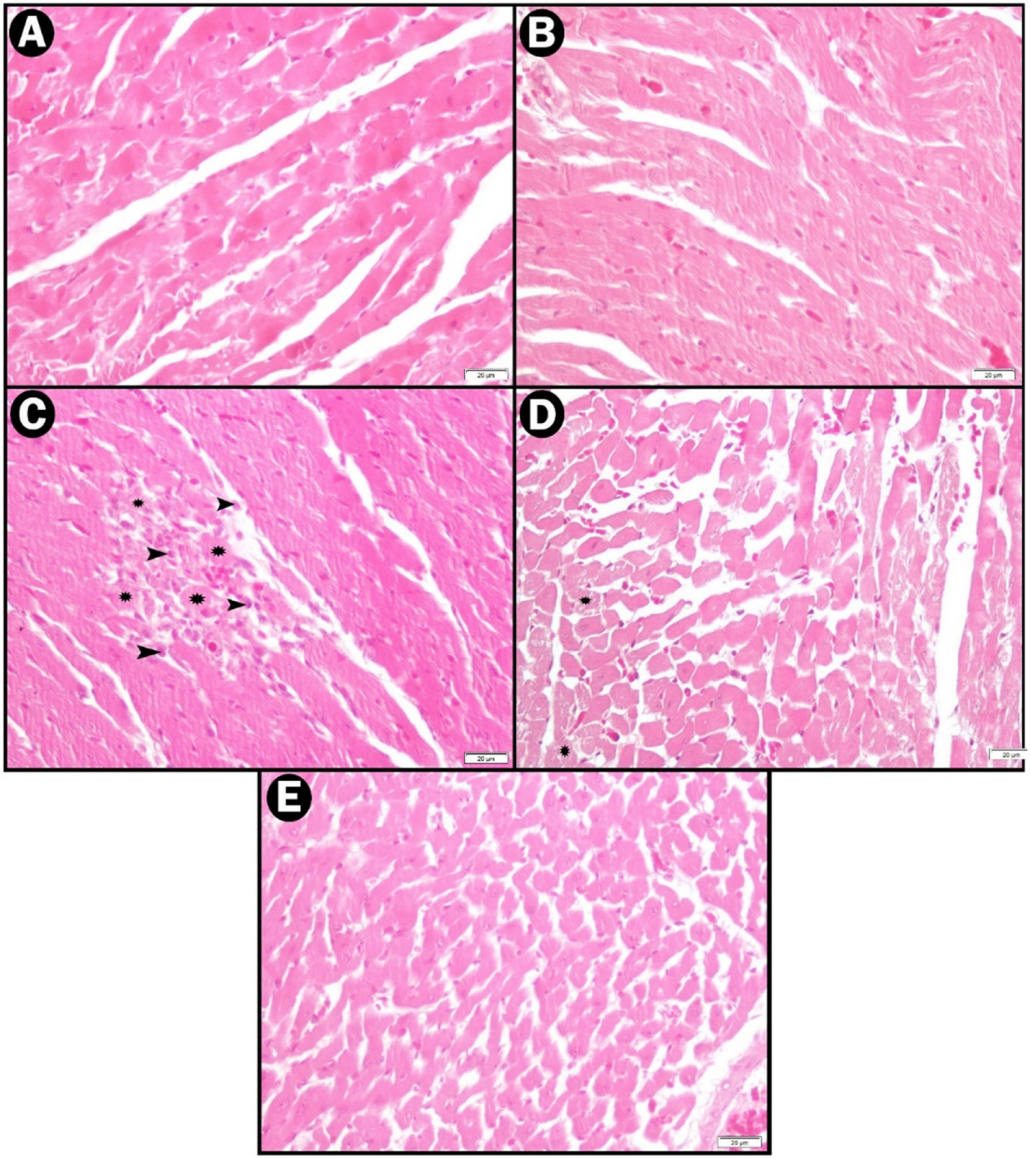
Histopatological findings (A) HEALTHY, (B) OXY 20, (C) ISO, (D) ISO + OXY 10, (E) ISO + OXY 20, arrows: inflammatory cell, stars: degenerated muscle fiber.

Based on the histopathological assessment, in the healthy group, cardiac muscle fibers appeared normal, with well-preserved connective tissue spaces between them (Figure 4A). Similarly, in the OXY 20 mg/kg group, the histological appearance closely resembled that of the healthy group, and no pathological abnormalities were evident (Figure 4B). Conversely, in the ISO-induced group, severe muscle fiber degeneration was observed, accompanied by a significant accumulation of inflammatory cells around the degenerated muscle cells (Figure 4C). In the ISO + OXY 10 mg/kg group, mild muscle fiber degeneration was noted, but there were no signs of inflammatory cell infiltration (Figure 4D). Remarkably, in the ISO + OXY 20 mg/kg group, no indications of muscle fiber degeneration or inflammation were detected, and the histological appearance resembled that of the healthy group (Figure 4E).

Histopathological findings were scored semiquantitatively for the intergroup evaluation according to the presence of degenerated muscle fibers and inflammatory cells. Accordingly, it was represented as absent or rare (−/+), mild (+), moderate (++), advanced (+++) (Table 2).

### Stereological Evaluation

**Figure 5.**
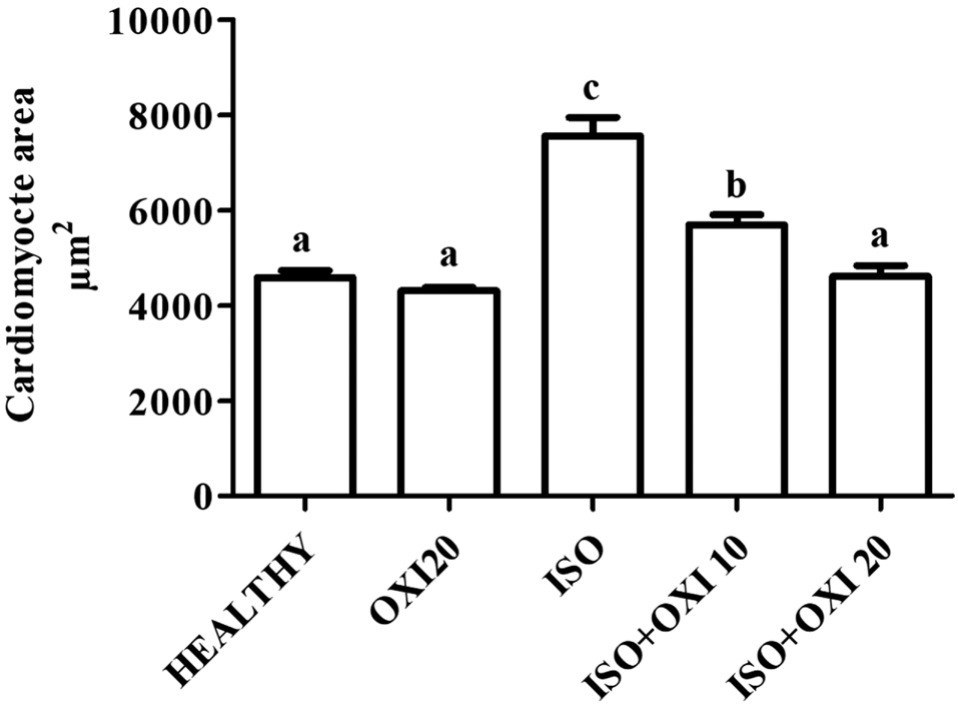
Cardiomyocyte area stereological evaluation findings.

The stereological nucleator technique and the One Way ANOVA Tukey multiple comparison test were used to compare the cardiomyocyte areas of the groups in the heart cross-section. The ISO and ISO + OXY 10 groups significantly differed from the healthy group (*P* < .05), but the ISO + OXY 20 and OXY 20 groups did not vary significantly from the healthy group (*P* > .05). (Figure 5).^27^

## Discussion

Strategies for the treatment of MI, which is one of the most prevalent and serious health problems because of high morbidity and mortality levels, have changed significantly during the last 2 decades. Therefore, the development of new therapeutic techniques including new drug and/or natural agent discovery against the occurrence of MI and the prevention of MI-related complications is a popular research area in the whole world.^28,7,29^ This study aimed to explore the potential cardioprotective effects of OXY, a widely recognized antioxidant agent, in a rat model of MI induced by ISO.

While numerous experimental animal models are available for MI studies, the ISO-induced MI model in rats stands out as an excellent choice due to its close resemblance to clinical conditions. ISO, a synthetic catecholamine, is particularly well-suited for this purpose. ISO exhibits effective beta-1 and beta-2 adrenergic receptor-blocking activity without affinity for alpha-adrenergic receptors. This unique pharmacological profile makes ISO a valuable tool for inducing MI in rats and replicating aspects of the clinical scenario. High doses of ISO, which is synthetic catecholamine, can affect myocardium tissue by increasing heart rate, mimicking acute MI and/or cardiotoxicity. Therefore, researchers generally prefer the standardized ISO-induced MI model in rats to determine the pleiotropic effects of potential cardioprotective drugs as future drug candidates.^8,30^

Cardiac TnI is a low molecular weight regulator. It is a myocardial protein that controls actin and myosin contraction via calcium ion-related interactions.^31^ during MI induced cell damage in heart tissue, increased levels of TnI are observed as a result of the extra release of this endogenous biomarker into the circulation. So it is accepted as a highly sensitive and specific marker for MI diagnosis.^32^ In our study, an increase in TnI levels was determined in the MI-control group of rats that received only ISO when compared to healthy controls. Khalil et al and Chen et al also suggested a similar increase in TnI levels after ISO-induced MI in rats.^6,32^ However, OXY administration caused a significant decrease in TnI levels at both doses when compared to ISO-treated rats. The high antioxidant property of OXY may be associated with this protective effect. By preventing oxidative damage to the heart muscle, the contractile function and structural integrity of the rat myocardium can be preserved. Another cardiac enzyme, CK-MB, is also an important biomarker of MI.^33^ The present study showed high CK-MB levels in ISO-induced MI group rats, in agreement with the study of Chen et al (2020).^34^ CK-MB levels were suppressed by OXY treatment, which significantly reduced myocardial damage.

Oxidative stress can be listed as the leading factor during the occurrence and pathophysiology of MI. The strong toxic effects of free oxygen radicals on heart tissue cannot be denied.^35^ Occurrence of immensely cardiotoxic free radicals such as superoxide, and also the end product of oxidative degradation of unsaturated fatty acids, MDA, contributes to MI-induced tissue damage. Namely, the MDA level which is an important biomarker of oxidative stress, increases in response to free radical generation in MI and is decreased by antioxidant systems during healing processes that are supported by antioxidant therapies.^36,37^

It has been reported that with the inhibition of the SOD enzyme due to oxidants in ischemic tissue during myocardial ischemia/reperfusion, the amount of GSH decreases, the amount of MDA increases, and the cells turn into a form that is less resistant to the toxic side effects of oxygen radicals.^7^ In our study, decreased SOD enzyme activity and GSH levels were observed in rats with an ISO-induced MI model. Conversely, the MDA level was significantly increased in the heart tissues of rats, which was also in line with previous reports.^7^ As seen in Figures 1-3, OXY (10 and 20 mg/kg) exerted favorable effects on antioxidant parameters in a dose-dependent manner. These results show that OXY reduces ROS and supports the antioxidant system in rats, in line with the previous literature.^36^ In a previously published article, the protective role of OXY were expressed.^38^ Supporting this study, our study demonstrated the protective role of OXY in cases of oxidative stress.

Histopathological findings obtained from Hematoxylin eosin staining for heart tissues are compatible with biochemical analyses. While mildly degenerated muscle fibers were determined in the ISO + OXY 10 group, no inflammatory cell accumulation was found in the ISO + OXY 20 group, and no evidence of degeneration or inflammation was found in the ISO + OXY 20 group, with an appearance similar to the healthy group being observed. On the other hand, severely degenerated muscle fibers and numerous clustered inflammatory cells around degenerated muscle cells were found in the myocardial tissues of animals given only ISO. Previous studies also support our present histopathological analyses.^28,7^ In addition, cardiomyocyte area was calculated using the stereological nucleator method in our study, and the results obtained support the histopathological findings. Accordingly, a significant difference between the ISO and ISO + OXY 10 groups and the healthy group was determined. Interestingly, the histopathological images obtained from OXY 10 and 20 mg/kg groups with ISO challenge were similar to those of the healthy rats.

There is insufficient data about the protective effects of OXY on cardiac tissue. With this study, it was found that OXY has a considerable protective and preventive potential against ISO-induced damage in myocardial cells. Although this study sheds light on OXY as a new potential natural therapeutic target for MI, further studies are necessary for the elucidation of the effect mechanism of OXY in reversing MI physiopathogenesis.

## Figures and Tables

**Table 1.** Effect of Oxyresveratrol on the CK-MB Activity and TnI Levels in Serum After Isoproterenol-Induced Myocardial Injury in Rats

Groups	CK-MB (U/L)	TnI (ng/mL)
Healthy	95,67 ± 11,46**^a^**	49,22 ± 5,80**^a^**
ISO control	394,18 ± 49,49**^c^**	309,47 ± 45,28**^c^**
OXY 20 mg/kg	115,92 ± 11,61**^a^**	56,57 ± 15,36**^a^**
ISO + OXY 10 mg/kg	271,77 ± 29,64**^b^**	211,28 ± 28,56**^b^**
ISO + OXY 20 mg/kg	251,89 ± 30,44**^b^**	197,97 ± 41,27**^b^**

ISO, isoproterenol; OXY, oxyresveratrol. Data are means ± SD. Columns indicated by different letters are significantly different (*P* < .05).

**Table 2.** Histopathological Scoring Between Groups

Groups	Degenerated Muscle Fıber	Inflammatory Cell
Healthy	−	−
OXY 20	−	−
ISO	++	++
ISO + OXY 10	+	−
ISO + OXY 20	−/+	−

ISO, isoproterenol; OXY, oxyresveratrol.

## Data Availability

The data that support the findings of this study are available on request from the corresponding author.
